# Can cultural capital, cognitive ability, and economic capacity help rural older adults bridge the digital divide? Evidence from an empirical study

**DOI:** 10.3389/fpubh.2024.1393651

**Published:** 2024-08-27

**Authors:** Yupeng Cui, Youshi He, Xinglong Xu, Lulin Zhou, Jonathan Aseye Nutakor

**Affiliations:** School of Management, Jiangsu University, Zhenjiang, China

**Keywords:** cultural capital, digital divide, rural older adults, cognitive ability, economic capacity

## Abstract

**Background:**

The digital divide is the difference between individuals who use the Internet and those who do not. Under the triple social environment of urban–rural dichotomy, population aging, and the digital era in China, the existence of digital divide among rural older adults has seriously affected their access to health information through the Internet, so it is urgent to bridge the digital divide problem they face.

**Methods:**

Based on Maslow’s Hierarchy of Needs Theory and Stress Coping Theory, the impact of cultural capital on the digital divide among rural older adults was systematically analyzed using hierarchical regression and Chained Mediation Effect Tests using data from the Chinese Family Panel Studies (CFPS).

**Results:**

Cultural capital has a significant positive effect on the digital divide among rural older adults (*β* = 0.178, *p* < 0.01). Cognitive ability and economic capacity both play independent intermediary effects between cultural capital and digital divide among rural older adults, and the intermediary chain formed by the two plays a chain intermediary effect. The increase in the cultural capital of rural older adults has led to an increase in their cognitive ability and economic capacity, which ultimately has a favorable effect on the bridging of the digital divide. Heterogeneity results suggest that cultural capital is more effective in bridging the digital divide among male rural older adults aged 60–69.

**Conclusion:**

Cultural capital is able to bridge the digital divide faced by rural older adults and is age and gender heterogeneous. At the same time, improved cognitive ability and economic capacity can also help rural older adults bridge the digital divide. Therefore, it is proposed that we increase the construction of public cultural service infrastructure in rural areas, liaise with community neighborhood committees and village committees to do a good job of publicity, improve training measures for key groups, and maintain the enthusiasm of rural older adults for learning, so as to provide references for the rural older adults in China and developing countries in general to bridge the digital divide.

## 1 Introduction

In recent years, China has been vigorously promoting the construction of digital infrastructure, and the continuous development and application of digital technology and digital products have laid a solid foundation for residents’ Internet accessibility (1). According to the data of the 51st Statistical Report on the Development of the Internet in China, as of December 2022, the number of Internet users in China has reached 1.067 billion, and the Internet penetration rate has reached 75.6%, of which the number of rural Internet users is only 308 million, accounting for 28.9% of the overall number of Internet users. The urban–rural dualization structure has long plagued China. The development speed of rural areas is far from catching up with the development speed of urban areas, and there is an obvious gap between urban and rural residents regarding their ability to access and use digital technology (2). At the same time, population aging is a major problem China must face. According to the National Bureau of Statistics, the number of older adults aged 60 years and above in China has reached 280 million, and the proportion of older adults aged 65 years and above is more than 14%, and the degree of aging is deepening. Older adults are confined to the objective reasons for declining physical function and the practical reasons for lower knowledge, skill, and digital literacy, making older adults struggle in the Internet era, and the status of older adults as a “digitally vulnerable group” has been further weakened. The digital divide is the difference between individuals who use the Internet and those who do not, and exists between countries, between urban and rural areas and even among older age groups (3). Internet is reshaping the original social structure and social relations by changing information access channels (4). Rural older adults are more prone to digital divide problems because of the backwardness of infrastructure construction in rural areas and the fact that rural older adults are far less educated, scientifically and culturally literate, and have far fewer social resources than their urban counterparts (5). The existence of the digital divide makes it problematic for older adults to access health information and utilize it. Therefore, in the triple context of the urban–rural dual structure, population aging and the digital age, helping older rural adults to bridge the digital divide among rural older adults is of positive significance for the implementation of the strategy of building digital villages, responding to the crisis of population aging and protecting the health of older adults.

In the Internet era, the primary basis for social stratification is the size of the ability to obtain and master information, and the smaller the ability to obtain and master information, the greater the digital divide problem will be the greater the problem of the digital divide (6). Some studies have shown that if the digital divide problem is not solved in time, it will lead to the further widening of the income gap, the frequent occurrence of social inequality, and even the deterioration of the physical and mental health of the residents and other serious consequences (7–9). The digital divide gap has become the fourth largest gap after the urban–rural gap, the agricultural-industrial gap, and the brainpower gap (10). Cultural capital has been shown to serve as a central mechanism for addressing social inequality, unequal distribution of resources, and health disparities among residents (11). Thus, cultural capital may have a positive impact on bridging the digital divide. Exploring the social group of rural older adults is of special significance, as Horolets et al. found that urban older adults tend to have higher levels of cultural capital than rural older adults (12). Although rural older adults are more dependent on traditional lifestyles, the marginal benefits of Internet use significantly impact rural older adults (13). Leguina et al. found that cultural capital can bridge people’s digital divide (14). Most existing studies separate cultural capital from the digital divide to explore its impact on health, income and other aspects, and only some scholars have investigated the relationship between cultural capital and the digital divide. Moreover, current research focuses less on the special group of rural older adults. Therefore, the study will conduct an in-depth study on the relationship between cultural capital and the digital divide among rural older adults.

From a theoretical point of view, cultural capital can be transformed into economic capital through certain ways to improve the economic capacity of residents (15). Income is extremely important for rural older adults. Most rural older adults do not have a stable source of income due to the limitations of knowledge, technology and other aspects. They cannot enjoy the benefits brought by the pension, and they have a single source of income, relying only on selling vegetables between the fields (16). According to Maslow’s hierarchy of needs theory, it is possible to pursue higher-level needs only when lower-level needs are satisfied. Education is generally considered as one of the manifestations of cultural capital. The population’s education level significantly affects the level of economic capacity; the higher the level of education, the higher the economic capacity (17). The investment in education brought by the increase in economic capacity will increase, motivating people to learn new knowledge and master new skills, thus bridging the digital divide gap.

Therefore, economic capacity may be an important mediating variable affecting cultural capital and the digital divide among rural older adults. In addition, bridging the digital divide gap is affected by external factors such as economic capacity and internal factors of rural older adults’ cognitive ability. Cognitive ability refers to how well an individual recognizes and grasps something (18). Cultural capital will positively affect the improvement of both the cognitive ability and non-cognitive ability of residents (19, 20), and the higher the educational level of residents, the stronger their cognitive ability will be. Enhanced cognitive ability will help rural older adults recognize the convenience that the Internet brings to their lives, prompting them to weaken their exclusion of Internet technology, thus bridging the digital divide gap they face. Therefore, cognitive ability may be another important mediating variable affecting cultural capital and the digital divide among rural older adults. Thus, the study incorporates cognitive ability and economic capacity into the analytical framework to explore the relationship between cultural capital and the digital divide among rural older adults.

Stress theory emphasizes that when residents feel pressure, they will take positive or negative measures to cope. The digital divide will pressure rural older adults in life, health, travel, etc. To better integrate into society, rural older adults need to meet social development needs by improving digital literacy. The study empirically analyzes the impact of cultural capital on the digital divide among rural older adults using data from the China Family Panel Studies (CFPS) and analyzes the impact of cultural capital on the digital divide among rural older adults by applying hierarchical regression and Bootstrap method, and explores the roles of cognitive ability and economic capacity in the impact of cultural capital on the digital divide among rural older adults, to reveal the mechanism of the impact of cultural capital on the digital divide among rural older adults.

## 2 Methods

### 2.1 Data sources

The China Family Panel Survey (CFPS) database is responsible by the China Social Science Survey Center of Peking University. It adopts a multi-stage, implicitly stratified, proportional systematic probability sampling method to survey China’s 25 provinces (autonomous regions and municipalities directly under the central government). The questionnaires consist of four parts: a questionnaire for children, a questionnaire for the community, a questionnaire for the family, and a questionnaire for the adult, which is representative of 95%. The CFPS data is selected for the study mainly based on the following two reasons: first, the data content involves the basic situation of rural older adults, family structure, medical insurance, living habits, etc., which can provide high-quality data support for this study; second, the data involves variables related to the research of this study, such as cultural capital, rural older adults’ Internet use status, etc., which is in line with the theme of the research of the study. The study is based on the China Family Panel Survey (CFPS) data, and rural older adults aged 60 and above are selected as the research object, and a total of 5,397 valid samples are obtained after invalid variables and missing variables are eliminated.

### 2.2 Variable description

#### 2.2.1 Dependent variable

The digital divide is a multidimensional aggregate. Drawing on the approach of Huang and Dou (21), the digital divide among rural older adults was categorized into three dimensions: the access gap, the usage gap and the information gap using CFPS data. The access gap is measured by the three questions of “whether they use mobile devices to access the Internet” and “whether they use computers to access the Internet,” and choosing “yes” implies that the digital divide is better bridged, the more likely it is that health information and resources are available online. The usage gap is measured by the continuous variables “In general, how often do you use your mobile device to access the Internet every day?” and “In general, how often do you use your computer to access the Internet every day” and the longer the time spent on the Internet, the better the effect of bridging the digital divide, the more likely it is that health information and resources are available online. The information gap is measured by nine items: “the importance of television as a means of accessing information,” “the importance of the Internet as a means of accessing information,” “the importance of radio as a means of accessing information,” “the importance of cell phone text messaging as a means of accessing information,” and “Importance of the Internet for work, leisure and recreation, keeping in touch with family and friends, study, daily life, “and the importance of the digital divide is better bridged by a scale of 1–5 according to the importance of the digital divide.

#### 2.2.2 Independent variable

The independent variable in this study is cultural capital. Because of the need for more objective cultural capital in the China Family Tracking Survey (CFPS) data, the study only analyzes cultural capital in terms of two dimensions, personality cultural capital and institutional cultural capital, to avoid data retention problems. Personality cultural capital mainly refers to the good temperament and habits formed by individuals and is measured by the three questions: “Have you read any books in the past 12 months?” Institutional cultural capital refers to an individual’s educational attainment and is measured by “Highest level of education completed,” which was transformed into the question “Have you received any education? The answer “No” corresponds to the original answer “Never attended school” and “Illiterate/semi-literate.” The answer “Yes” corresponds to the original answer “Elementary school,” “Junior high school,” “High school/Junior high school/Technical school/Vocational high school,” “Three-year college,” “Undergraduate,” “Master,” “Doctor.”

#### 2.2.3 Mediating variable

The mediating variables in this study are cognitive ability and economic capacity. For the measurement of cognitive ability, the question “How much of the main things that happened to you in a week can you remember?” was used as an indicator of cognitive ability (22). The higher the memory, the higher the cognitive ability of rural older adults. Economic capacity was measured regarding wage income, pension and children’s support to rural older adults (23).

#### 2.2.4 Control variable

In The study, variables such as basic personal characteristics (gender, age), living habits (smoking, drinking), and medical insurance which may have a greater impact on the dependent variable (digital divide among rural older adults) and the mediating variables (cognitive ability, economic capacity), are controlled to obtain more reliable test results. Variable assignments and descriptive statistics are shown in Table 1.

**Table 1:** Variable assignment and descriptive statistics.

Variables	Dimension	Description of item or variable	Variable assignment	M	SD
Digital divide	Access gap	Whether they use mobile devices to access the Internet	Yes = 1, No = 0	0.647	0.173
		Whether they use computers to access the Internet	Yes = 1, No = 0		
	Usage gap	How often do you use your mobile device to access the Internet every day?	Add the two and take the logarithm	0.291	0.103
		How often do you use your computer to access the Internet every day?			
	Information gap	Importance of television, Internet, radio, and cell phone text messaging as a means of accessing information	Scale of importance from 1 to 5, with 1 being very unimportant and 5 being very important	2.331	0.445
		Importance of the Internet for work, leisure and recreation, keeping in touch with family and friends, study, daily life			
Cultural capital	Personalized cultural capital	Have you read any books in the past 12 months	Yes = 1, No = 0	0.084	0.278
	Institutional cultural capital	Have you received any education	Yes = 1, No = 0	0.510	0.500
Mediating variable	Cognitive ability	How much of the main things that happened to you in a week can you remember	Remember only a little = 1, remember only a few = 2, remember half = 3, remember most = 4, remember completely = 5	2.360	1.298
	Economic capacity	Wage income, pension and children’s support	Add the three and take the logarithm	2.415	1.144
Control variable	Personal characteristics	Gender	Male = 1，Female = 0	0.470	0.499
		Age	60–69 years = 1, 70–79 years = 2, 80–89 years = 3, 90–99 years = 4	1.390	0.526
	Medical insurance	Whether enrolled in medical insurance	Yes = 1, No = 0	0.910	0.290
	Living habits	Smoke	Yes = 1, No = 0	0.310	0.462
		Drink	Yes = 1, No = 0	0.150	0.362

### 2.3 Correlation analysis

To initially test the relationship between the variables, the study conducts correlation analysis, and the results are shown in Table 2. The correlation coefficients between cultural capital, cognitive ability, economic capacity and digital divide among rural older adults are all significantly correlated at the 1% level.

**Table 2:** Results of correlation analysis.

Variables	1	2	3	4	5	6	7	8	9	10
1: Gender										
2: Age	−0.021									
3: Medical Insurance	−0.059^***^	−0.022								
4: Smoke	−0.523^***^	−0.023	0.017							
5: Drink	−0.319^***^	0.0.061^***^	0.003	0.232^***^						
6: Personalized cultural capital	−0.166^***^	0.004	0.031^**^	0.052^***^	0.084^***^					
7: Institutional cultural capital	−0.352^***^	−0.095^***^	0.056^***^	0.154^***^	0.092^***^	0.208^***^				
8: Cultural capital	−0.361^***^	−0.086^***^	0.061^***^	0.151^***^	0.108^***^	0.616^***^	0.938^***^			
9: Cognitive ability	−0.141^***^	−0.004	0.050^***^	0.086^***^	0.096^***^	0.143^***^	0.142^***^	0.175^***^		
10: Economic capacity	−0.124^***^	−0.058^***^	0.048^***^	0.064^***^	0.073^***^	0.088^***^	0.094^***^	0.116^***^	0.090^***^	
11: Digital divide	−0.161^***^	−0.162^***^	0.051^***^	0.085^***^	0.072^***^	0.148^***^	0.248^***^	0.292^***^	0.129^***^	0.095^***^

^*^, ^**^, and ^***^ are significant at the 10, 5, and 1% levels (two-sided), respectively.

### 2.4 Statistical analysis

This study used SPSS 22.0 with STATA 16.0 to collate the entered data and descriptive statistics to analyze the basic personal characteristics of rural older adults. Hierarchical regression and chained mediation effect tests were used to analyze the impact of cultural capital on the digital divide among rural older adults and the mechanisms by which cognitive ability and economic capacity play a role in it. Endogeneity and robustness tests were conducted using PSM and replacement variable methods, respectively. Finally, heterogeneity is analyzed using split-sample regression. Differences were considered statistically significant at *p* < 0.1.

## 3 Results

The results of the multicollinearity test show that each model’s variance inflation factor (VIF) value is less than 2, and the tolerance between cultural capital, cognitive ability and economic capacity is above 0.6. Therefore, there is no serious multicollinearity between the selected variables in this study.

### 3.1 Cultural capital and the digital divide among rural older adults

Table 3 shows the results of the hierarchical regression test. The Model 1 and Model 2 in Table 3 show the results of the impact of cultural capital on the digital divide among rural older adults. Model 1 only puts in the control variables, and Model 2 adds the independent variable, cultural capital, based on Model 1. Model 2 shows that cultural capital has a significant positive effect on the digital divide among rural older adults (*β* = 0.178, *p* < 0.01), which indicates that the higher the level of cultural capital of rural older adults, the better they can bridge the digital divide and the smaller the digital divide problem they face. At the same time, after adding the independent variable cultural capital, the R^2^ of model 2 increases by 0.044 compared with model 1, which indicates that cultural capital has a stronger explanatory power for the digital divide among rural older adults.

**Table 3:** Hierarchical regression test results.

Variables	Digital divide among rural older adults				Cognitive ability	Economic capacity
	Model 1	Model 2	Model 3	Model 4	Model 5	Model 6
Cultural capital		0.178^***^ (0.011)	0.169^***^ (0.011)	0.175^***^ (0.011)	0.613^***^ (0.060)	0.275^***^ (0.053)
Cognitive ability			0.015^***^ (0.003)			
Economic capacity				0.011^***^ (0.003)		
Gender	−0.078^***^ (0.008)	−0.036^***^ (0.008)	−0.033^***^ (0.008)	−0.033^***^ (0.008)	−0.149^***^ (0.044)	−0.196^***^ (0.039)
Age	−0.077^***^ (0.006)	−0.069^***^ (0.006)	−0.069^***^ (0.006)	−0.067^***^ (0.006)	0.016 (0.033)	−0.118^***^ (0.029)
Medical insurance	0.032^***^ (0.011)	0.026^**^ (0.011)	0.023^**^ (0.011)	0.024^**^ (0.011)	0.166^***^ (0.060)	0.147^***^ (0.053)
Smoke	−0.004 (0.008)	0.003 (0.008)	0.002 (0.008)	0.003 (0.008)	0.058 (0.044)	−0.005 (0.039)
Drink	0.017^*^ (0.010)	0.016^*^ (0.009)	0.013 (0.009)	0.015 (0.009)	0.204^***^ (0.051)	0.120^***^ (0.045)
R^2^	0.055	0.099	0.104	0.101	0.043	0.027
Adjusted R^2^	0.054	0.098	0.103	0.100	0.042	0.026
F	63.192^***^	98.283^***^	89.684^***^	86.506^***^	40.647^***^	24.746^***^
N	5,396	5,396	5,396	5,396	5,396	5,396

^*^, ^**^, and ^***^ are significant at the 10, 5, and 1% levels, respectively; heteroskedasticity robust standard errors are in parentheses.

### 3.2 Mediating effects of cognitive ability

Referring to the mediating effect test method proposed by Wen and Ye (24), Model 3 and Model 5 in Table 3 show the test results of the mediating effect of cognitive ability between cultural capital and digital divide among rural older adults. In Model 5, cultural capital has a positive effect on the cognitive ability of rural older adults (*β* = 0.613, *p* < 0.01). Model 3 positively affects cognitive ability on the digital divide among rural older adults after adding the mediating variable cognitive ability (*β* = 0.015, *p* < 0.01). At the same time, the influence coefficient of cultural capital decreases from 0.178 in Model 2 to 0.169 in Model 3, indicating that cognitive ability partially mediates the process of cultural capital influencing the digital divide among rural older adults. To address the problem of low testing power, the study adopts the Bootstrap method to conduct further tests, as shown in Table 4. The indirect effect of cognitive ability has a confidence interval of [0.006, 0.013] at the 95% level, which does not contain 0, indicating a partially mediated effect of cognitive ability. In the paths of personality cultural capital-cognitive ability-digital divide among rural older adults and institutional cultural capital-cognitive ability-digital divide among rural older adults, the confidence intervals do not include 0, indicating that cognitive ability has partial mediating effects in the process of influencing the digital divide among rural older adults in all dimensions of cultural capital.

**Table 4:** Results of the mediation effect test of cognitive ability based on Bootstrap methodology.

Path	Effect	Bootstrap S.E.	95%CI	
			LL	UL
Direct effect				
Cultural capital-digital divide among rural older adults	0.169	0.011	0.147	0.191
personalized cultural capital-digital divide among rural older adults	0.099	0.012	0.076	0.122
Institutional cultural capital-digital divide among rural older adults	0.094	0.007	0.081	0.108
Indirect effect				
Cultural capital-cognitive ability-digital divide among rural older adults	0.009	0.002	0.006	0.013
personalized cultural capital-cognitive ability-digital divide among rural older adults	0.010	0.002	0.006	0.014
Institutional cultural capital-cognitive ability-digital divide among rural older adults	0.005	0.001	0.003	0.007

Bootstrap intervals are estimated as repeated self-sampling 1,000 times 95% confidence intervals.

### 3.3 Mediating effects of economic capacity

Model 4 and Model 6 in Table 3, show the test results of the mediating effect of economic capacity between cultural capital and digital divide among rural older adults. In Model 6, cultural capital has a significant positive effect on the cognitive ability of rural older adults (*β* = 0.275, *p* < 0.01). In model 4, economic capacity significantly positively affects the digital divide among rural older adults (*β* = 0.010, *p* < 0.01), indicating that rural older adults with high economic capacity can better bridge the digital divide. Meanwhile, the regression coefficient of cultural capital decreases from 0.178 in Model 2 to 0.175 in Model 4, indicating that economic capacity partially mediates cultural capital and digital divide among rural older adults. The Bootstrap method is still used for further testing, as shown in Table 5. The indirect effect of economic capacity has a confidence interval of [0.001, 0.005] at the 95% level, which does not contain 0, indicating a partial mediating effect of economic capacity. After splitting cultural capital according to dimensions, the interval estimation results show that the mediating effect of economic capacity still passes the significance test, indicating that economic capacity has a partial mediating effect in all dimensions of cultural capital, affecting the digital divide among rural older adults.

**Table 5:** Results of the mediation effect test of economic capacity based on Bootstrap methodology.

Path	Effect	Bootstrap S.E.	95%CI	
			LL	UL
Direct effect				
Cultural capital-digital divide among rural older adults	0.175	0.011	0.153	0.197
personalized cultural capital-digital divide among rural older adults	0.106	0.012	0.083	0.129
Institutional cultural capital-digital divide among rural older adults	0.098	0.007	0.084	0.111
Indirect effect				
Cultural capital-economic capacity-digital divide among rural older adults	0.003	0.001	0.001	0.005
personalized cultural capital-economic capacity-digital divide among rural older adults	0.003	0.001	0.001	0.006
Institutional cultural capital-economic capacity-digital divide among rural older adults	0.001	0.001	0.001	0.003

Bootstrap intervals are estimated as repeated self-sampling 1,000 times 95% confidence intervals.

### 3.4 Chain mediation effect test

The bootstrap method was used to test the chain mediating effects involved in this paper, and the results are shown in Table 6. The confidence interval at the 95% level for the mediating effects of cognitive ability and economic capacity is [0.0001, 0.0006], excluding 0. Specifically, the influence of cultural capital on the digital divide among rural older adults is subject to three indirect effects, and all of them reach a significant level. First, the mediating effect value in the path of the cultural capital-cognitive ability-digital divide is 0.0087, with a confidence interval of [0.0054, 0.0123] at the 95% level, excluding 0. Second, the mediating effect value in the cultural capital-cognitive ability-economic capacity-digital divide pathway is 0.0003, with a confidence interval of [0.0001, 0.0006] at the 95% level, which does not contain zeros. Third, the mediation effect in the path from cultural capital-economic capacity-digital divide has a value of 0.0023, with a confidence interval at the 95% level of [0.0007, 0.0046], which does not contain 0. The results of the interval estimation after splitting cultural capital based on its dimensions show that the chained mediation effect of cognitive ability and economic capacity still passes the significance test.

**Table 6:** Results of the chained mediation effect test of cognitive ability and economic capacity based on Bootstrap approach.

Path	Effect	Bootstrap S.E.	95%CI	
			LL	UL
1. Total indirect effect of cultural capital on the digital divide	0.0113	0.0021	0.0075	0.0158
Cultural capital-cognitive ability-digital divide among rural older adults	0.0087	0.0018	0.0054	0.0123
Cultural capital-cognitive ability-economic capacity-digital divide among rural older adults	0.0003	0.0001	0.0001	0.0006
Cultural capital-economic capacity-digital divide among rural older adults	0.0023	0.0010	0.0007	0.0046
2. Total indirect effect of personality cultural capital on the digital divide	0.0125	0.0022	0.0086	0.0172
personalized cultural capital-cognitive ability-digital divide among rural older adults	0.0095	0.0018	0.0062	0.0134
personalized cultural capital-cognitive ability-economic capacity-digital divide among rural older adults	0.0003	0.0001	0.0001	0.0006
personalized cultural capital-economic capacity-digital divide among rural older adults	0.0027	0.0011	0.0009	0.0050
3. Total indirect effects of institutional cultural capital on the digital divide	0.0056	0.0011	0.0037	0.0080
Institutional cultural capital-cognitive ability-digital divide among rural older adults	0.0044	0.0009	0.0028	0.0065
Institutional cultural capital-cognitive ability-economic capacity-digital divide among rural older adults	0.0002	0.0001	0.0001	0.0003
Institutional cultural capital-economic capacity-digital divide among rural older adults	0.0011	0.0005	0.0003	0.0023

Bootstrap intervals are estimated as repeated self-sampling 1,000 times 95% confidence intervals.

### 3.5 Endogeneity and robustness tests

#### 3.5.1 Endogeneity tests

The digital divide among rural older adults in China is affected by cultural capital, cognitive ability and economic capacity, and thus, bridging the digital divide among rural older adults in China is not merely the result of random assignment. To eliminate the endogeneity of the model as much as possible, this paper chooses the propensity score matching method (PSM). Taking the cultural capital owned by rural older adults as an example, because cultural capital is a discrete variable in this paper, it needs to be treated as a dichotomous variable to determine the control group and the treatment group; therefore, according to the distribution of the score of the cultural capital in the sample, the score of 0 will be categorized as “low cultural capital,” and the score of 1 and 2 will be categorized as “high cultural capital.” At the same time, the probability of the digital divide among rural older adults in China was calculated, i.e., the propensity value. The treatment group of “high cultural capital” and the control group of “low cultural capital” were matched according to the size of the propensity value to estimate ATT (average treatment effect on the treated) of cultural capital on the digital divide among rural older adults.

In this paper, we choose three matching methods, K-nearest neighbor matching, radius matching and kernel matching, to be analyzed to avoid the bidirectional causal effect of cultural capital on the digital divide among rural older adults. The test results are shown in Table 7. The ATT of *K*-nearest neighbor matching, radius matching and kernel matching are all significant at the 1% level, and the study shows that cultural capital has a significant effect on the digital divide among rural older adults after controlling for selection bias, which indicates that the positive effect of cultural capital on the digital divide among rural older adults is robust and potentially less endogenous risk after excluding selection bias.

**Table 7:** The ATT effect of cultural capital on the digital divide among rural older adults.

Variables	Matching methods	ATT	S.E.	*t*
Cultural capital	*K*-nearest neighbor	0.195^***^	0.042	4.59
	Radius	0.123^***^	0.006	21.81
	Kernel	0.107^***^	0.007	15.38

^*^, ^**^, and ^***^ are significant at the 10, 5, and 1% levels, respectively; heteroskedasticity robust standard errors are in parentheses.

#### 3.5.2 Robustness test

The robustness test of the replacement variable is chosen for this study. This paper uses the access gap as a replacement variable for the digital divide among rural older adults for regression. For the convenience of controlled observation, the regression is presented uniformly with the previous paper (Table 8). The robustness test results are basically the same as the regression results, indicating that the regression results of this paper are quite robust.

**Table 8:** Robustness test results.

Variables	Access gaps among rural older adults			
	Model 7	Model 8	Model 9	Model 10
Cultural capital		0.115^***^ (0.008)	0.110^***^ (0.008)	0.108^***^ (0.008)
Cognitive ability			0.008^***^ (0.002)	0.008^***^ (0.002)
Economic capacity				0.008^***^ (0.002)
Gender	−0.032^***^ (0.006)	−0.004 (0.006)	−0.003 (0.006)	−0.001 (0.006)
Age	−0.052^***^ (0.006)	−0.046^***^ (0.004)	−0.047^***^ (0.004)	−0.046^***^ (0.004)
Medical insurance	0.017^**^ (0.008)	0.013^*^ (0.008)	0.012 (0.008)	0.011 (0.008)
Smoke	0.007 (0.006)	0.012^**^ (0.006)	0.011^*^ (0.006)	0.011^*^ (0.006)
Drink	0.003 (0.007)	0.003 (0.006)	0.001 (0.007)	0.001 (0.007)
R^2^	0.037	0.073	0.077	0.080
Adjusted R^2^	0.036	0.072	0.076	0.078
F	41.339^***^	71.189^***^	64.201^***^	58.171^***^
N	5,396	5,396	5,396	5,396

^*^, ^**^, and ^***^ are significant at the 10, 5, and 1% levels, respectively; heteroskedasticity robust standard errors are in parentheses.

### 3.6 Heterogeneous analysis of cultural capital on the digital divide among rural older adults

#### 3.6.1 Robustness test

Table 9 shows the results of the test for gender heterogeneity and age heterogeneity. As can be seen in Table 9, cultural capital has a positive effect on the digital divide among rural older adults for males (*β* = 0.195, *p* < 0.01), and cultural capital has the same positive effect on the digital divide among rural older adults for females (*β* = 0.170, *p* < 0.01).

**Table 9:** Heterogeneity test results of cultural capital on digital divide among rural older adults.

Variables	Male	Female	Low age group	Middle age group	High age group
Cultural capital	0.195^***^ (0.015)	0.170^***^ (0.017)	0.240^***^ (0.016)	0.074^***^ (0.014)	0.152^***^ (0.057)
Control variables	Yes	Yes	Yes	Yes	Yes
R^2^	0.100	0.071	0094	0.048	0.126
Adjusted R^2^	0.098	0.069	0.093	0.045	0.079
F	56.261^***^	43.339^***^	69.814^***^	19.367^***^	2.662^***^

^*^, ^**^, and ^***^ are significant at the 10, 5, and 1% levels, respectively.

#### 3.6.2 Robustness test

The age group “60–69” is redefined as a “low age group,” “70–79” as a “middle age group,” and “80–99” as a “high age group.” As seen in Table 9, cultural capital positively affects the digital divide among rural older adults in the low age group (*β* = 0.240, *p* < 0.01). Cultural capital positively affects the digital divide among rural older adults in the middle age group (*β* = 0.074, *p* < 0.01). Cultural capital positively affects the digital divide among rural older adults in the high age group (*β* = 0.152, *p* < 0.01).

## 4 Discussion

### 4.1 Cultural capital positively influences the bridging of the digital divide among rural older adults

The study found that cultural capital can positively influence the digital divide among rural older adults, which is similar to the findings of Cui et al. (6). The higher the level of cultural capital among rural older adults, the better they can bridge the digital divide and the more likely it is that health information and resources will be available online. Cultural capital, as a core mechanism for solving the problems of social inequality, unequal distribution of resources, and health disparities, can have an impact on bridging the digital divide among rural older adults in two ways. First, the accumulation of personality cultural capital is mainly cultivated through cultural activities such as attending concerts, watching cultural performances, and watching movies. On the one hand, individuals can transform the good artistic culture they receive into their unique temperament by participating in cultural activities, and studies have shown that immersive virtual environments can help individuals stimulate their desire to learn and enhance their comprehension ability (25). By listening to music, watching TV dramas and listening to news on the Internet, rural older adults can improve their enthusiasm for going online and enhance their confidence in taking the initiative to learn Internet information.

On the other hand, although personality cultural capital cannot be directly transferred to others, it can also be transformed into social capital through special ways, thus promoting the dissemination of information among different groups (26). The “gossip center” at the village entrance is generally an important place for rural older adults to obtain information, where they can grasp a variety of information, which, to a certain extent, also promotes the transformation of cultural capital into social capital. When some rural older adults realize the importance of the Internet and mention the importance of “chat centers,” it often has a significant positive impact on the popularization of the Internet, which also plays a positive role in bridging the digital divide among rural older adults. Second, institutional cultural capital is mainly manifested in the various certificates obtained. The more certificates obtained, the better education an individual has received. The higher the level of education, the higher the qualifications obtained, the more receptive to new things, and the stronger the positive initiative to learn. Some studies have shown that educational attainment also significantly impacts poverty alleviation (27). Poverty is the main cause of poor Internet access in rural areas, and education can significantly improve the situation. Furthermore, as children live outside all the time, the Internet becomes the most convenient way for rural older adults to contact their children, which will also motivate older adults to try to use online communication tools, thus alleviating the problem of digital divide among rural older adults to a certain extent.

### 4.2 Cognitive ability has a mediating effect in the way cultural capital influences the digital divide among rural older adults

The study found that cognitive ability has a mediating effect in the process of cultural capital influencing the digital divide among rural older adults and that cognitive ability has helped rural older adults broaden their horizons and recognize the importance of the Internet in today’s society. Improving rural older adults’ cultural capital led to improving their cognitive ability and better bridging the digital divide among rural older adults. Cognitive ability is the basis for individuals’ courage to try unknown things and the prerequisite for individuals to recognize the world. Research has found that individuals with high cognitive ability tend to have better physical health, memory ability, reaction ability, and work performance (28). Internet use is closely related to an individual’s cognitive ability, and individuals with higher cognitive ability are more willing to use the Internet because they can realize the significant changes that the Internet has brought to life in today’s society. The higher the cognitive ability of an individual, the greater the possibility of recognizing the world. Making rural older adults realize the importance of the Internet in contacting their children and increasing their income is important in stimulating their learning motivation. Cultural capital positively impacts the cognitive ability of rural older adults, similar to the findings of Kai’s study (29). Rural older adults improve their cultural capital by watching movies, listening to musicals, visiting museums, science and technology centers and other cultural activities. Rural older adults also broaden their horizons and increase their knowledge by participating in these activities, improving their cognitive ability to a certain extent. From this, the improvement of cognitive ability can significantly influence the cultural capital level of rural older adults and bridge the digital divide among rural older adults.

### 4.3 Economic capacity has a mediating effect in the way cultural capital influences the digital divide among rural older adults

The study found that economic capacity mediates cultural capital, influencing the digital divide among rural older adults and that economic capacity provides a material guarantee for rural older adults to learn and use the Internet. An increase in the level of cultural capital of rural older adults will facilitate the improvement of their economic capacity, and the digital divide among rural older adults will be better bridged. The level of economic capacity determines an individual’s quality of life and the social stratum in which an individual lives. From the institutional level, digital village construction is a political system implemented by the state to increase farmers’ income and realize common prosperity. The basis of digital village construction is industrial digitization, and using the Internet to digitally transform agriculture and manufacturing is one of the basic requirements of digital village construction (30). While the Internet empowers the development of rural productivity, it can, to a certain extent, promote the synergistic governance of labor, land and other factors of production, thus playing an important role in rural economic development and promoting farmers’ income (31). Zhang and other studies show that digital village construction significantly affects farmers’ income in less developed areas, while the impact on the income of urban residents could be more satisfactory (32). The improvement of farmers’ economic capacity will counteract the development of digital technology in rural areas, and the better the degree of development of digital technology in rural areas, the higher the degree of rural older adults’ exposure to it, the greater the possibility of bridging the digital divide. From the cultural capital perspective, cultural capital is the basic condition for individuals to participate in all social activities and come into contact with different class groups. It is also one of the preconditions for improving economic capacity. Consumption level can reflect a family’s economic strength from the side, and the direction of residents’ consumption often reflects a family’s interests. Generally, the stronger the consumption ability of residents for cultural products, the more cultural capital stock they have (33). Rural older adults are limited by their own cultural limitations, it is difficult to expand their source of income, in addition to planting crops, but also rely on their children to give, but most of the rural older adults are reluctant to accept their children’s belongings for the sake of face. Therefore, the level of cultural capital of rural older adults has an impact on their economic capacity.

### 4.4 Cognitive ability and economic capacity have a chain mediating effect in the way cultural capital influences the digital divide among rural older adults

Different from previous studies, our study found that cognitive ability and economic capacity would play a chain mediating role in the process of cultural capital influencing the digital divide among rural older adults, and cognitive ability is a prerequisite for rural older adults to learn Internet technology, and a key element to improving the economic capacity of rural older adults. The vast majority of rural older adults in China have a low level of education and have not formed a systematic cultural habitus, which makes it challenging to learn and master the Internet, as well as to standardize the identification of Internet information (34). Some studies have shown that the improvement of cognitive ability can bring about the improvement of economic capacity (35). If rural older adults can realize the importance of the Internet and take the initiative to learn Internet knowledge, the more helpful it will be to improve their economic capacity. Some scholars have confirmed that cognitive ability mediates the effect of Internet use on residents’ income (36). In addition, the results of Chen et al. showed that cognitive ability can improve farmers’ economic capacity (37). It can be seen that rural older adults improve their level of cultural capital through acquired learning or participation in cultural activities, based on which cognitive ability is enhanced, which in turn increases the income of rural older adults and ultimately prompts older adults to learn, master, and utilize the Internet technology, to achieve the effect of helping rural older adults bridge the digital divide among rural older adults.

### 4.5 Gender heterogeneity and age heterogeneity in the impact of cultural capital on the digital divide among rural older adults

From the results of gender heterogeneity, cultural capital has a greater impact on the digital divide among rural older adults than among rural older women. This may be because rural older adults are more seriously influenced by the traditional idea of “emphasizing children over daughters,” and women are less likely to study education in traditional societies, which significantly inhibits the contribution of cultural capital to their digital divide. Interestingly, there is no uniform conclusion about the gender digital divide in the academic world. Some scholars believe that with the popularization of the Internet and the decline in the cost of available devices, the gender digital divide is disappearing with the increase in age, and the younger the age, the less pronounced the gender digital divide is, but this kind of view has been criticized by some scholars (38). In analyzing the digital divide faced by teenagers from seven countries in Europe, South America and Australia, Masanet et al. found that the gender digital divide still exists in the younger generation, and female digital competence is lower than male digital competence (39). From the results of age heterogeneity, the impact of cultural capital on the digital divide among rural older adults is greatest for rural older adults aged 60–69 years old, followed by rural older adults aged 80 years old and above, and smallest for rural older adults aged 70–79 years old, showing a “U-shaped” relationship. This is because rural older adults aged 60–69 should have enjoyed retirement and received pensions, but due to the pressure of life, they have to choose to continue to struggle, so their desire for Internet technology is higher, and their enthusiasm for learning is also higher. For rural older adults aged 80 and above, the older they are, the more dependent they are on their loved ones and the stronger their sense of loneliness is, so Internet technology can help them quickly contact their loved ones and get spiritual solace (40). As for rural older adults aged 70–79, their children can already be on their own, which, coupled with the deterioration of their physical functions, causes them to slow down the pace of life and enjoy their family life in peace.

### 4.6 Strengths and limitations

This study has several strengths. For example, this study used the most recently released data, selected a large sample size, and the findings can be generalized to relevant populations. The study’s results also examined factors such as cultural capital, cognitive ability, and economic capacity, enabling a comprehensive assessment of the relationship between these factors and the digital divide among rural older adults. However, this study also has some limitations. For example, the cross-sectional design used in this study was unable to capture the causal relationships between the variables accurately, nor was it able to understand the temporal relationships between the variables; a longitudinal study would be more appropriate in subsequent studies. In addition, the mechanism of influencing the digital divide among rural older adults is more complex and involves a number of factors. In contrast, the study is only a preliminary exploration under limited conditions and should be explored in greater depth in future studies.

## 5 Conclusion

The study found that, first, cultural capital has a significant positive effect on the digital divide among rural older adults, and the higher the level of cultural capital of rural older adults, the easier it is for them to bridge the digital divide and the more likely it is that health information and resources are available online. Second, cognitive ability and economic capacity have independent mediating roles in the influence of cultural capital on the digital divide among rural older adults. Cognitive ability enables rural older adults to broaden their horizons and recognize the importance of the Internet; economic capacity provides material security for rural older adults to learn and use the Internet, thus continuously bridging the digital divide among rural older adults. Third, cognitive ability and economic capacity play a chain mediating role in the influence of cultural capital on the digital divide among rural older adults. By continuously improving the level of cultural capital, the cognitive ability and economic capacity of rural older adults are improved, thus bridging the digital divide among rural older adults. Fourth, cultural capital is more effective in bridging the digital divide among rural older adults who are male and aged 60–69. This suggests that attention should be paid to cultivating the cultural capital of rural older adults and improving their cognitive ability and economic capacity, thus providing a reference for bridging the digital divide among rural older adults and encouraging them to use the Internet to access health information.

The possible marginal contributions of the study are: first, to analyze the path to bridge the digital divide gap from the cultural capital perspective of rural older adults to enrich the antecedent research on the digital divide. Second, the intrinsic factor of cognitive ability and the extrinsic factor of economic capacity are included in the analytical framework, and the hierarchical regression and Bootstrap method are adopted to empirically explore the influence of cultural capital on the digital divide among older adults as well as the mediating roles of cognitive ability and economic capacity, to enrich the research related to the digital divide issue. Third, against the triple background of the urban–rural dichotomy, population aging and the digital age, we take a sample of 5,397 rural older adults in China as a research sample and reveal the positive role of cultural capital in bridging the digital divide among rural older adults. It provides empirical evidence for improving the level of cultural capital of rural older adults, bridging the digital divide faced by rural older adults, building a digital countryside, coping with the crisis of population aging and protecting the health of older adults.

The findings of this study also have important policy implications. They suggest that attention should be paid to the construction of infrastructure for rural public cultural services. First, it is necessary to ensure that public cultural services are accessible, that the resources for the provision of rural public cultural services can meet the appropriate standards, and that rural public cultural services can be accepted and used by the public to meet the spiritual and cultural needs of rural older adults. Secondly, we should pay attention to the rural public cultural services can play the role of transformation to ensure the economic interests of rural older adults. Take the Moganshan B&B in Zhejiang as an example, creating a folklore library based on local characteristics, attracting travelers from all over the country to come to play (41). This makes local young people return to their hometowns to start their businesses and improves rural older adults’ income. They also show that the role of the government should be fully emphasized, and the government should link the community neighborhood committee and village committee to increase the investment in publicity and improve the training work. On the one hand, the government should invest more in publicity, using a combination of online and offline methods. Online, it should use popular APPs such as Jitterbug and Shutterbug, as well as WeChat public numbers, and offline, it should organize lectures and other forms of publicity on the importance of the Internet to promote the development of the agricultural economy. On the other hand, training is constantly being improved, especially for this key group of people who do not have pensions and rely on growing crops for economic income. In addition, it is important to give full play to the role of the family to stimulate the enthusiasm of older adults to learn. The Internet can bring older adults closer to their loved ones and positively impact the improvement of economic income. In order to adapt to the ever-developing requirements of society, older adults have to try to use the Internet, and children helping older adults to learn the Internet are more likely to be accepted by older adults, which can play a pivotal role in bridging the digital divide among rural older adults, thus forming a government-driven, community-guaranteed, family-supported, personal efforts of a good learning situation. The findings of the study can provide implications for rural older adults in developing countries in general to improve their cultural capital and contribute to bridging the digital divide.

## Data Availability

The original contributions presented in the study are included in the article/Supplementary material, further inquiries can be directed to the corresponding author.
