# Supplementary material for: Can cultural capital, cognitive ability, and economic capacity help rural older adults bridge the digital divide? Evidence from an empirical study
Source: Front Public Health. 2024 Aug 27;12:1393651. doi: 10.3389/fpubh.2024.1393651 (PMC11385861; doi:10.3389/fpubh.2024.1393651)
Supplement: Supplementary file 1 [file Data_Sheet_1.pdf]

Supplementary Material

1 Supplementary Tables

TABLE 1 Variable assignment and descriptive statistics.

Variables	Dimension	Description of item or variable	Variable assignment	M	SD
Digital divide	Access gap	whether they use mobile devices to access the Internet	Yes=1, No=0	0.647	0.173
		whether they use computers to access the Internet	Yes=1, No=0		
	Usage gap	How often do you use your mobile device to access the Internet every day?	Add the two and take the logarithm	0.291	0.103
		How often do you use your computer to access the Internet every day?			
	Information gap	Importance of television, Internet, radio, and cell phone text messaging as a means of accessing information	Scale of importance from 1 to 5, with 1 being very unimportant and 5 being very important	2.331	0.445
		Importance of the Internet for work, leisure and recreation, keeping in touch with family and friends, study, daily life			
Cultural capital	Personalized cultural capital	Have you read any books in the past 12 months	Yes=1, No=0	0.084	0.278
	Institutional cultural capital	Have you received any education	Yes=1, No=0	0.510	0.500
Mediating variable	Cognitive ability	How much of the main things that happened to you in a week can you remember	Remember only a little=1, remember only a few=2, remember half=3, remember most=4, remember completely=5	2.360	1.298
	Economic capacity	Wage income, pension and children's support	Add the three and take the logarithm	2.415	1.144
Control variable	Personal characteristics	Gender	Male=1，Female=0	0.470	0.499
		Age	60-69 years=1, 70-79 years=2, 80-89 years=3, 90-99 years=4	1.390	0.526
	Medical insurance	Whether enrolled in medical insurance	Yes=1, No=0	0.910	0.290

Living habits	Smoke	Yes=1, No=0	0.310	0.462
	Drink	Yes=1, No=0	0.150	0.362

TABLE 2 Results of correlation analysis.

Variables	1	2	3	4	5	6	7	8	9	10
1: gender										
2: age	-0.021									
3: medical insurance	-0.059***	-0.022								
4: smoke	-0.523***	-0.023	0.017							
5: drink	-0.319***	0..061***	0.003	0.232***						
6: personalized cultural capital	-0.166***	0.004	0.031**	0.052***	0.084***					
7: Institutional cultural capital	-0.352***	-0.095***	0.056***	0.154***	0.092***	0.208***				
8: cultural capital	-0.361***	-0.086***	0.061***	0.151***	0.108***	0.616***	0.938***			
9: cognitive ability	-0.141***	-0.004	0.050***	0.086***	0.096***	0.143***	0.142***	0.175***		
10: economic capacity	-0.124***	-0.058***	0.048***	0.064***	0.073***	0.088***	0.094***	0.116***	0.090***	
11: digital divide	-0.161***	-0.162***	0.051***	0.085***	0.072***	0.148***	0.248***	0.292***	0.129***	0.095***

Note: *, **, and *** are significant at the 10%, 5%, and 1% levels (two-sided), respectively.

TABLE 3 Hierarchical regression test results.

Variables	Digital divide among rural older adults				Cognitive ability	Economic capacity
	Model 1	Model 2	Model 3	Model 4	Model 5	Model 6
Cultural capital		0.178***	0.169***	0.175***	0.613***	0.275***
		(0.011)	(0.011)	(0.011)	(0.060)	(0.053)
Cognitive ability			0.015***			
			(0.003)			
Economic capacity				0.011***		
				(0.003)		
Gender	-0.078***	-0.036***	-0.033***	-0.033***	-0.149***	-0.196***
	(0.008)	(0.008)	(0.008)	(0.008)	(0.044)	(0.039)
Age	-0.077***	-0.069***	-0.069***	-0.067***	0.016	-0.118***
	(0.006)	(0.006)	(0.006)	(0.006)	(0.033)	(0.029)
Medical insurance	0.032***	0.026**	0.023**	0.024**	0.166***	0.147***
	(0.011)	(0.011)	(0.011)	(0.011)	(0.060)	(0.053)
Smoke	-0.004	0.003	0.002	0.003	0.058	-0.005
	(0.008)	(0.008)	(0.008)	(0.008)	(0.044)	(0.039)
Drink	0.017*	0.016*	0.013	0.015	0.204***	0.120***
	(0.010)	(0.009)	(0.009)	(0.009)	(0.051)	(0.045)
R^2	0.055	0.099	0.104	0.101	0.043	0.027
Adjusted R^2	0.054	0.098	0.103	0.100	0.042	0.026
F	63.192***	98.283***	89.684***	86.506***	40.647***	24.746***
N	5396	5396	5396	5396	5396	5396

Note: *, **, and *** are significant at the 10%, 5%, and 1% levels, respectively; heteroskedasticity robust standard errors are in parentheses.

TABLE 4 Results of the mediation effect test of cognitive ability based on Bootstrap methodology.

Path	Effect	Bootstrap S.E.	95%CI	
			LL	UL
Direct effect				
Cultural capital-digital divide among rural older adults	0.169	0.011	0.147	0.191
personalized cultural capital-digital divide among rural older adults	0.099	0.012	0.076	0.122

Institutional cultural capital-digital divide among rural older adults	0.094	0.007	0.081	0.108
Indirect effect				
Cultural capital-cognitive ability-digital divide among rural older adults	0.009	0.002	0.006	0.013
personalized cultural capital-cognitive ability-digital divide among rural older adults	0.010	0.002	0.006	0.014
Institutional cultural capital-cognitive ability-digital divide among rural older adults	0.005	0.001	0.003	0.007

Note: Bootstrap intervals are estimated as repeated self-sampling 1000 times 95% confidence intervals.

TABLE 5 Results of the mediation effect test of economic capacity based on Bootstrap methodology.

Path	Effect	Bootstrap S.E.	95%CI	
			LL	UL
Direct effect				
Cultural capital-digital divide among rural older adults	0.175	0.011	0.153	0.197
personalized cultural capital-digital divide among rural older adults	0.106	0.012	0.083	0.129
Institutional cultural capital-digital divide among rural older adults	0.098	0.007	0.084	0.111
Indirect effect				
Cultural capital-economic capacity-digital divide among rural older adults	0.003	0.001	0.001	0.005
personalized cultural capital-economic capacity-digital divide among rural older adults	0.003	0.001	0.001	0.006
Institutional cultural capital-economic capacity-digital divide among rural older adults	0.001	0.001	0.001	0.003

Note: Bootstrap intervals are estimated as repeated self-sampling 1000 times 95% confidence intervals.

TABLE 6 Results of the chained mediation effect test of cognitive ability and economic capacity based on Bootstrap approach.

Path	Effect	Bootstrap S.E.	95%CI	
			LL	UL
1. Total indirect effect of cultural capital on the digital divide	0.0113	0.0021	0.0075	0.0158
Cultural capital-cognitive ability-digital divide among rural older adults	0.0087	0.0018	0.0054	0.0123
Cultural capital-cognitive ability-economic capacity-digital divide among rural older adults	0.0003	0.0001	0.0001	0.0006
Cultural capital-economic capacity-digital divide among rural older adults	0.0023	0.0010	0.0007	0.0046
2. Total indirect effect of personality cultural capital on the digital divide	0.0125	0.0022	0.0086	0.0172
personalized cultural capital-cognitive ability-digital divide among rural older adults	0.0095	0.0018	0.0062	0.0134
personalized cultural capital-cognitive ability-economic capacity-digital divide among rural older adults	0.0003	0.0001	0.0001	0.0006
personalized cultural capital-economic capacity-digital divide among rural older adults	0.0027	0.0011	0.0009	0.0050
3. Total indirect effects of institutional cultural capital on the digital divide	0.0056	0.0011	0.0037	0.0080
Institutional cultural capital-cognitive ability-digital divide among rural older adults	0.0044	0.0009	0.0028	0.0065
Institutional cultural capital-cognitive ability-economic capacity-digital divide among rural older adults	0.0002	0.0001	0.0001	0.0003
Institutional cultural capital-economic capacity-digital divide among rural older adults	0.0011	0.0005	0.0003	0.0023

Note: Bootstrap intervals are estimated as repeated self-sampling 1000 times 95% confidence intervals.

TABLE 7 The ATT effect of cultural capital on the digital divide among rural older adults.

Variables	Matching methods	ATT	S.E.	t
Cultural capital	*K*-nearest neighbor	0.195^{***}	0.042	4.59
	Radius	0.123^{***}	0.006	21.81
	Kernel	0.107^{***}	0.007	15.38

Note: *, **, and *** are significant at the 10%, 5%, and 1% levels, respectively; heteroskedasticity robust standard errors are in parentheses.

TABLE 8 Robustness test results.

Variables	Access gaps among rural older adults			
	Model 7	Model 8	Model 9	Model 10
Cultural capital		0.115^{***}	0.110^{***}	0.108^{***}
		(0.008)	(0.008)	(0.008)
Cognitive ability			0.008^{***}	0.008^{***}

			(0.002)	(0.002)
Economic capacity				0.008***
				(0.002)
Gender	-0.032***	-0.004	-0.003	-0.001
	(0.006)	(0.006)	(0.006)	(0.006)
Age	-0.052***	-0.046***	-0.047***	-0.046***
	(0.006)	(0.004)	(0.004)	(0.004)
Medical insurance	0.017**	0.013*	0.012	0.011
	(0.008)	(0.008)	(0.008)	(0.008)
Smoke	0.007	0.012**	0.011*	0.011*
	(0.006)	(0.006)	(0.006)	(0.006)
Drink	0.003	0.003	0.001	0.001
	(0.007)	(0.006)	(0.007)	(0.007)
R^2	0.037	0.073	0.077	0.080
Adjusted R^2	0.036	0.072	0.076	0.078
F	41.339***	71.189***	64.201***	58.171***
N	5396	5396	5396	5396

Note: *, **, and *** are significant at the 10%, 5%, and 1% levels, respectively; heteroskedasticity robust standard errors are in parentheses.

TABLE 9 Heterogeneity test results of cultural capital on digital divide among rural older adults.

Variables	Male	Female	Low age group	middle age group	High age group
Cultural capital	0.195***	0.170***	0.240***	0.074***	0.152***
	(0.015)	(0.017)	(0.016)	(0.014)	(0.057)
Control variables	Yes	Yes	Yes	Yes	Yes
R^2	0.100	0.071	0094	0.048	0.126
Adjusted R^2	0.098	0.069	0.093	0.045	0.079
F	56.261***	43.339***	69.814***	19.367***	2.662***

Note: *, **, and *** are significant at the 10%, 5%, and 1% levels, respectively.
